# Molecular Detection of Tick-Borne Pathogens in Humans with Tick Bites and Erythema Migrans, in the Netherlands

**DOI:** 10.1371/journal.pntd.0005042

**Published:** 2016-10-05

**Authors:** Setareh Jahfari, Agnetha Hofhuis, Manoj Fonville, Joke van der Giessen, Wilfrid van Pelt, Hein Sprong

**Affiliations:** Centre for Infectious Disease Control, National Institute for Public Health and the Environment (RIVM), Bilthoven, the Netherlands; Baylor College of Medicine, UNITED STATES

## Abstract

**Background:**

Tick-borne diseases are the most prevalent vector-borne diseases in Europe. Knowledge on the incidence and clinical presentation of other tick-borne diseases than Lyme borreliosis and tick-borne encephalitis is minimal, despite the high human exposure to these pathogens through tick bites. Using molecular detection techniques, the frequency of tick-borne infections after exposure through tick bites was estimated.

**Methods:**

Ticks, blood samples and questionnaires on health status were collected from patients that visited their general practitioner with a tick bite or erythema migrans in 2007 and 2008. The presence of several tick-borne pathogens in 314 ticks and 626 blood samples of this cohort were analyzed using PCR-based methods. Using multivariate logistic regression, associations were explored between pathogens detected in blood and self-reported symptoms at enrolment and during a three-month follow-up period.

**Results:**

Half of the ticks removed from humans tested positive for *Borrelia burgdorferi* sensu lato, *Anaplasma phagocytophilum*, *Candidatus* Neoehrlichia mikurensis, *Rickettsia helvetica*, *Rickettsia monacensis*, *Borrelia miyamotoi* and several *Babesia* species. Among 92 *Borrelia burgdorferi* s. l. positive ticks, 33% carried another pathogen from a different genus. In blood of sixteen out of 626 persons with tick bites or erythema migrans, DNA was detected from *Candidatus* Neoehrlichia mikurensis (n = 7), *Anaplasma phagocytophilum* (n = 5), *Babesia divergens* (n = 3), *Borrelia miyamotoi* (n = 1) and *Borrelia burgdorferi* s. l. (n = 1). None of these sixteen individuals reported any overt symptoms that would indicate a corresponding illness during the three-month follow-up period. No associations were found between the presence of pathogen DNA in blood and; self-reported symptoms, with pathogen DNA in the corresponding ticks (n = 8), reported tick attachment duration, tick engorgement, or antibiotic treatment at enrolment.

**Conclusions:**

Based on molecular detection techniques, the probability of infection with a tick-borne pathogen other than Lyme spirochetes after a tick bite is roughly 2.4%, in the Netherlands. Similarly, among patients with erythema migrans, the probability of a co-infection with another tick-borne pathogen is approximately 2.7%. How often these infections cause disease symptoms or to what extend co-infections affect the course of Lyme borreliosis needs further investigations.

## Author Summary

Two most common tick-borne diseases in Europe are Lyme borreliosis and tick-borne encephalitis. Ticks transmit many more pathogens, causing neglected diseases such as anaplasmosis, babesiosis, rickettsiosis and neoehrlichiosis. These diseases are seldom diagnosed, due to their mild and non-characteristic symptoms, but also due to lack of awareness and availability of diagnostic tests. Using molecular detection techniques (polymerase chain reaction or PCR), we estimated the frequency of tick-borne infections in humans after a tick bite and in patients with the first symptoms of Lyme borreliosis, an erythema migrans. About half of the ticks that fed on humans carried one or more tick-borne pathogens, and approximately 2.5% of people that were bitten by ticks were infected with a tick-borne pathogen other than Lyme borreliosis or tick-borne encephalitis. Co-infections of a tick-borne pathogen in patients with an erythema migrans was also approximately 2.5%. Based on these findings, we estimated the incidence of tick-borne infections other than Lyme borreliosis in the Netherlands. How often these infections cause disease or to what extend co-infections affect the course of Lyme borreliosis needs further investigations.

## Introduction

Lyme borreliosis is the most prevalent tick-borne disease in humans, and is caused by spirochetes of the *Borrelia burgdorferi* sensu lato complex [1–3]. The most common clinical manifestation of early localized Lyme borreliosis is erythema migrans (EM), an expanding skin lesion occurring after several days or weeks at the site of the tick bite. Other sporadically reported symptoms in this early stage of disease are malaise and viral-like symptoms. Disseminated Lyme borreliosis displays more severe manifestations that can involve a patient’s nervous system, joints, skin, and in rare cases the heart [1–3]. Tick-borne encephalitis (TBE) is the most common tick-borne central nervous system infection caused by the tick-borne encephalitis virus (TBEV). Its clinical spectrum ranges from fever to mild meningitis and severe meningoencephalitis with or without paralysis [4].

In several European countries, there have been marked increases in the incidence of Lyme borreliosis and TBE over the past ten to twenty years [5–7]. In the Netherlands, a retrospective study among general practitioners has shown a continuing increase in consultations for tick bites and EM between 1994 and 2009 [8, 9]. The increasing number of tick bites, adding up to 1.1 million tick bites in 2009 [8], poses a growing risk of disseminated Lyme borreliosis and perhaps also of other tick-borne diseases. In the Netherlands, *Ixodes ricinus* ticks transmit several *Borrelia burgdorferi* s. l. genospecies, but are also infected with a variety of established or potentially pathogenic microorganisms, such as *Borrelia miyamotoi*, *Anaplasma phagocytophilum*, *Candidatus* Neoehrlichia mikurensis, several *Babesia* species, *Rickettsia helvetica*, *R*. *monacensis* and TBEV [10–15]. These ticks often carry multiple pathogens; at least one-third of the *I*. *ricinus* ticks carrying *B*. *burgdorferi* s. l. are co-infected with one or more pathogens from a different genus [12], implying frequent exposure and possibly subsequent infection with several pathogens when humans are bitten by ticks.

Remarkably, little is known about the incidences and clinical presentations of other tick-borne diseases than Lyme borreliosis and TBE. In general, disease caused by these other tick-borne pathogens, are associated with febrile illnesses with fever, headache, myalgia and malaise [16–21]. However, in immunocompromised patients chronic infections with severe clinical manifestations and even mortality have been described [17, 18, 21, 22]. In the Netherlands, one single case of anaplasmosis has been reported in 1999 [23], and one case of *B*. *miyamotoi* disease in an immunocompromised patient in 2012 [18]. It has been suggested that the severity of disease in Lyme borreliosis is affected by co-infections with other tick-borne pathogens [24–28]. Therefore, co-infections of *B*. *burgdorferi* s. l. with different tick-borne pathogens may possibly contribute to the high variety of clinical manifestations that are associated with Lyme borreliosis.

Several reasons can be appointed for the absence in reporting of tick-borne diseases other than Lyme borreliosis and TBE, and the diagnosis of co-infections with other pathogens in Lyme borreliosis patients. Firstly, most of these infections might be self-limiting without overt or characteristic symptoms, often a clear-cut case definition of patients infected with one of these pathogens has not been established yet. Secondly, a poor performance or non-existence of supportive laboratory tests in routine medical microbiological settings. Thirdly, the lack of awareness among health professionals.

Here, we aim to investigate i) whether infection with tick-borne pathogens other than *B*. *burgdorferi* s. l. can be shown in patients with early localized Lyme borreliosis and in people exposed to tick bites in the Netherlands, and to determine ii) the clinical picture of patients with DNA of tick-borne pathogens in their blood.

Our approach is to test for the presence of nucleic acid (DNA/RNA) of the specific pathogens in human blood through amplification with PCR, especially since currently; there is no other specific laboratory diagnostic to detect infection with most of these tick-borne pathogens. Compared to DNA amplification with PCR, available serological tests generally have a low specificity and or sensitivity, particularly during the early phase of infection. In addition, although culturing is considered the most reliable method in proving the presence of microorganisms, it is time consuming, costly and often not possible for all pathogens.

## Materials and Methods

### Study design, ticks, human samples and questionnaires

Ticks, EDTA-blood and questionnaire data were available from a nationwide prospective observational study among patients who consulted one of 307 enrolling general practitioners for a tick bite or EM between May 2007 and December 2008 in the Netherlands, as described in detail [29]. All participants gave written informed consent, all minors who participated in the study had consent given from a parent/guardian, and the study protocol (number 07-032/K) was approved by the medical ethics committee of the University Medical Centre in Utrecht, the Netherlands. Patients were not eligible for participation when they were younger than six years of age, or when the tick bite had occurred outside the Netherlands. At enrolment, participants received the first set of study materials, containing a brochure about the study, an enrolment questionnaire, and materials for collection and mailing of first blood samples and removed ticks. Ticks removed from the skin were submitted in a small tube with 70% ethanol. In total, 314 ticks were obtained from 293 participants, of which 278 patients consulted their physician for a tick bite, and fifteen patients consulted their physician with an EM. Four ticks (1%) were larvae, 167 (53%) nymphs, 135 (43%) adult ticks, and for eight ticks, the developmental stage could not be determined, as they had been damaged too much during removal from the patient’s skin. No other tick species than *I*. *ricinus* were identified. At enrolment, two tubes of blood were collected, 7 ml in a serum tube and 5 ml in an EDTA tube. Three months after enrolment, follow-up questionnaires and a consecutive 7 ml serum sample was collected from the tick bitten patients and from the EM patients after standard antibiotic treatment [29, 30]. Seven patients who consulted their physician for a tick bite and in whom EM developed within the three month follow-up duration of the prospective study, were categorized in EM patient-group of the current study. EDTA-blood samples were available for molecular testing from 335 tick bitten patients and 291 EM patients.

### Tick analyses for detection of tick-borne pathogens

After arrival at the laboratory, ticks were stored at –20°C in ethanol. DNA was extracted using the DNeasy Blood and Tissue Kit (Qiagen, Hilden, Germany) according to the manufacturer’s instructions extraction for ticks. After total DNA extraction from ticks and amplification by PCR, reverse line blotting (RLB) was performed for *Borrelia*-, *Ehrlichia*-, *Anaplasma*-, *Rickettsia*- and *Babesia*-species. Further identification by DNA sequencing was performed as described [11, 31]. PCR products that specifically reacted to the generic (“catch all”) probes, but that could not be further specified to the (geno) species level were designated as “untypeable”. Furthermore, our RLB analysis could not distinguish between *B*. *garinii* and *B*. *bavariensis* [12]. The presence of *B*. *miyamotoi* in ticks was tested by a real-time PCR amplification in 302 ticks, and *Candidatus* Neoehrlichia mikurensis in 312 ticks. The presence of TBEV RNA could not be screened in the tick samples, since only DNA had been extracted from these samples. Individual test results of the tick analyses were not reported to the participants or their physicians, in accordance with the informed consent form.

### Molecular analyses for detection of tick-borne pathogens in EDTA-blood

Extraction of whole nucleic acid of the EDTA-blood samples were performed using robot-extraction (MagNA Pure Compact Extraction Robot; Roche, Basel, Switzerland) from 400 μL of EDTA-plasma (Nucleic Acid Isolation Kit I; Roche) according to the manufacturer’s instructions in a diagnostic laboratory setting. All 626 samples were analyzed with different real-time PCRs based on various genes specific for the microorganism of interest namely; *B*. *burgdorferi* s. l., *B*. *miyamotoi*, *A*. *phagocytophilum*, *Candidatus* Neoehrlichia mikurensis, spotted fever *Rickettsia*'s carried out on a LightCycler 480 (Roche Diagnostics Nederland B.V, Almere, the Netherlands). For primers and probes, see S1 Table (supplementary data). Reactions were done in a final volume of 20 μl with iQ multiplex Powermix, 3 μl of sample and 0.2 μM for all primers and different concentrations for probes. Positive plasmid controls and negative water controls were used on every plate tested. For detection of TBEV, multiplex a reverse transcription real-time PCR was performed as described before [32]. In brief, reactions were done in a final volume of 20 μl with TaqMan Fast Virus 1-Step Master Mix (Thermo Fisher scientific, USA), 5 μl of sample and 0.2 μM for all primers and 0.2 μM probes (S1 Table) were added to the master mix and internal control was added to all the samples. With 20 min reverse transcription step at 50°C, denaturation at 95°C for 30 s and 50 cycles of 95°C for 10 s and 60°C for 30 s. The amplification was performed on a Roche LightCycler 480 instrument. For *Babesia* genospecies, we performed a conventional PCR targeting the *18S rRNA* gene on all the blood samples [11], followed by sequencing. To minimize cross contamination and false-positive results, negative controls were included in each batch tested by PCR. In addition, DNA/RNA extraction, PCR mix preparation, sample addition, and PCR analyses were performed in separated air locked dedicated labs. On all samples that were found positive in the real-time PCR, conventional PCRs were performed for confirmation on one or more targets followed by Tris-Borate-EDTA-agarose gel-electrophoresis. PCR products were sequenced, and these were compared with reference sequences from Genbank using Unweighted Pair Group Method with Arithmetic Mean-based (UPGMA) hierarchical clustering. Individual test results of these molecular analyses on EDTA-blood were not reported to the participants or their physicians, in accordance with the informed consent form.

### Statistical analyses

The prevalence of microorganism DNA detection in ticks and in EDTA-blood was calculated with 95% confidence intervals (95%CI) based on mid-P exact. Characteristics of persons with or without DNA detected in blood by PCR were compared in Chi-square or Fisher’s exact test. We looked for associations between DNA detected in EDTA-blood by PCR and DNA detected in available ticks from the participants, tick engorgement, patient-reported tick attachment duration, antibiotic treatment at enrolment, and patient-reported symptoms at enrolment and after three months. Using multivariate logistic regression, we explored for associations between DNA detected in blood by PCR and self-reported symptoms at enrolment and follow-up. All reported clinical symptoms (at enrolment and follow-up) were included as predictive variables in the multivariate logistic regression models, after which the models were optimized using backwards elimination, until all predictive variables that were maintained in the model were statistically significant contributors (p<0.05). Statistical analyses were performed with SAS 9.4 (SAS Inc.).

## Results

### Tick-borne pathogens in ticks removed from humans

Table 1 shows the number of DNA sequences of the pathogens detected in 314 ticks obtained from 293 participants. *Borrelia burgdorferi* s. l. DNA was detected in 92 (29%) ticks, as published earlier [29]. The ticks contained DNA of *Candidatus* Neoehrlichia mikurensis (5.4%), *A*. *phagocytophilum* (1.0%), *Rickettsia* species (22%), *Babesia* species (3.5%). and *B*. *miyamotoi* (2.3%). DNA of microorganisms of two or more genera were detected in 34 ticks (11%). Among the 92 *B*. *burgdorferi* s. l. positive ticks, 30 (33%) also carried a pathogen of a different genus. About half of the ticks (149/314, 47%) tested negative for all genera.

**Table 1:** Detected DNA sequences in 314 ticks obtained from 293 participants. The results on *B*. *burgdorferi* s. l. have been published by Hofhuis et al. 2013 [29].

Detected DNA sequences		n	*/ N*	*%*	*(95%CI)*	Estimated human exposure with 1.1 million tick bites
*Borrelia burgdorferi* sensu lato [29]		92	*/ 314*	*29*.*3%*	*(24*.*5%-34*.*5%)*	322293
	*B*. *afzelii* [29]	36	*/ 314*	*11*.*5%*	*(8*.*3%-15*.*4%)*	126115
	*B*. *garinii* [29]	11	*/ 314*	*3*.*5%*	*(1*.*9%-6*.*0%)*	38535
	*B*. *burgdorferi* senso stricto [29]	7	*/ 314*	*2*.*2%*	*(1*.*0%-4*.*4%)*	24522
	*B*. *valaisiana* [29]	4	*/ 314*	*1*.*3%*	*(0*.*4%-3*.*0%)*	14013
	Untypeable* *Borrelia burgdorferi* [29]	36	*/ 314*	*11*.*5%*		
*Borrelia miyamotoi*		7	*/ 302*	*2*.*3%*	*(1*.*0%-4*.*5%)*	25497
*Babesia* spp		11	*/ 314*	*3*.*5%*	*(1*.*8%-6*.*0%)*	
	*B*. *microti*	6	*/ 314*	*1*.*9%*	*(0*.*8%-3*.*9%)*	21019
	*B*. *venatorum (B*. *EU1)*	4	*/ 314*	*1*.*3%*	*(0*.*4%-3*.*0%)*	14013
	*B*. *divergens*	1	*/ 314*	*0*.*3%*	*(0*.*0%-1*.*6%)*	3503
*Ehrlichia* spp / *Anaplasma* spp		8	*/ 314*	*2*.*5%*	*(1*.*2%-4*.*8%)*	
	*A*. *phagocytophilum*	3	*/ 314*	*1*.*0%*	*(0*.*2%-2*.*6%)*	10510
	Untypeable* *Ehrlichia* / *Anaplasma* spp	5	*/ 314*	*1*.*6%*		
*Candidatus* Neoehrlichia mikurensis		17	*/ 312*	*5*.*4%*	*(3*.*3%-8*.*4%)*	59936
Spotted fever rickettsia’s		70	*/ 314*	*22*.*3%*	*(18*.*0%-27*.*2%)*	
	*R*. *helvetica*	59	*/ 314*	*18*.*8%*	*(14*.*8%-23*.*4%)*	206688
	*R*. *monacensis*	1	*/ 314*	*0*.*3%*	*(0*.*0%-1*.*6%)*	3503
	Untypeable* *Rickettsia* spp	10	*/ 314*	*3*.*2%*		
Co-infections with *B*. *burgdorferi* sensu lato**		30				105096
	*Babesia* spp	3	*/ 314*	*1*.*0%*	*(0*.*2%-2*.*6%)*	
	*Ehrlichia* / *Anaplasma* spp	10	*/ 314*	*3*.*2%*	*(1*.*6%-5*.*6%)*	
	*Candidatus* Neoehrlichia mikurensis					
	Spotted fever rickettsia’s	21	*/ 314*	*6*.*7%*	*(4*.*3%-9*.*9%)*	
	*Borrelia miyamotoi*	1	*/ 302*	*0*.*3%*	*(0*.*0%-1*.*6%)*	

Using the observed prevalence of tick-borne pathogens in 314 ticks, national annual numbers of human exposure were estimated among 1.1 million tick bites in the Netherlands [8].

* PCR products that specifically reacted to the generic (“catch all”) probes, but that could not be further specified to the (geno) species level were designated. as Untypeable. Within *B*. *burgdorferi* s. l., RLB analysis could not distinguish between *B*. *garinii* and *B*. *bavariensis* [29].

** These categories of co-infections with *B*. *burgdorferi* s. l. are not mutually exclusive.

### Tick-borne pathogens in human EDTA-blood

Table 2 shows the prevalence of DNA detection of tick-borne pathogens in EDTA-blood samples of 335 tick bitten patients and 291 EM patients, using various (real-time) PCRs. Only one (0.2%) of 626 blood samples tested positive for *B*. *burgdorferi* s. l. and one (0.2%) for *B*. *miyamotoi* in the real-time PCRs multiplex, both with high Ct values. In another multiplex, five blood samples (0.8%) were positive for *A*. *phagocytophilum* and seven (1.1%) for *Candidatus* Neoehrlichia mikurensis. Three (0.5%) blood samples for *Babesia* genospecies yielded a sequence in conventional PCR, in which genetic analyses showed to be *B*. *divergens*. None of the samples were found positive for spotted fever Rickettsia's or TBEV.

**Table 2:** Prevalence of DNA detection of tick-borne pathogens in blood of persons with tick bites or erythema migrans (EM), as determined by PCRs.

	EM patients (n = 291)		Tick bitten patients (n = 335)		Total (n = 626)		Estimated number of infection among 1.1 million tick bites
	n	*%*	n	*%*	n	*% (95%CI)*	n
*Borrelia burgdorferi* s. l.	1	*0*.*3%*	0	*-*	1	*0*.*2% (0*.*0%–0*.*8%)*	1757
*Borrelia miyamotoi*	1	*0*.*3%*	0	-	1	*0*.*2% (0*.*0%–0*.*8%)*	1757
*Anaplasma phagocytophilum*	2	*0*.*7%*	3	*0*.*9%*	5	*0*.*8% (0*.*3%–1*.*8%)*	8786
*Candidatus* Neoehrlichia mikurensis	4	*1*.*4%*	3	*0*.*9%*	7	*1*.*1% (0*.*5%–2*.*2%)*	12300
*Babesia divergens*	1	*0*.*3%*	2	*0*.*6%*	3	*0*.*5% (0*.*1%–1*.*3%)*	5272
Spotted fever *Rickettsia* species	0	-	0	-	0	*0*.*0% (0*.*0%–0*.*5%)*	-
Tick-borne encephalitis virus	0	-	0	-	0	*0*.*0% (0*.*0%–0*.*5%)*	-
Total (excluding *B*. *burgdorferi* s. l.)	8	*2*.*7%*	8	*2*.*4%*	16	*2*.*6% (1*.*5%–4*.*0%)*	28115

EDTA blood samples testing were tested in various (real-time) PCRs for the presence of tick-borne pathogens. Using the observed prevalence of infection with tick-borne pathogens, national numbers of infections per year were estimated among 1.1 million tick bites in the Netherlands [8]. Note that the prevalence of DNA confirmed *Borrelia burgdorferi* s. l. detection in blood is a small fraction of the number of manifest borreliosis cases. For explanation, see results section. 95% CI = 95% confidence intervals.

**Fig 1:**
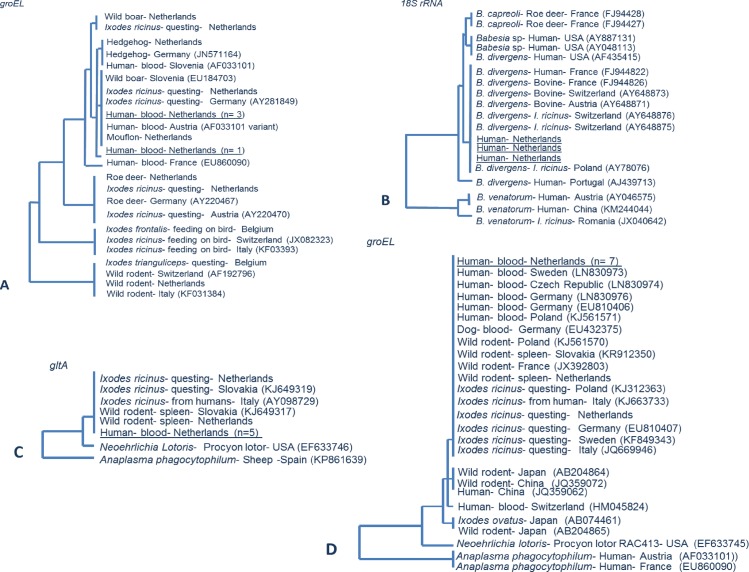
Phylogenetic tree of the sequences obtained from human blood samples. PCR and sequencing was performed on the real-time PCR-positive blood samples. Sequences were clustered with (reference) sequences from Genbank. The evolutionary distance values were determined by Kimura method, and the tree was constructed according to the neighbor-joining method. A.) *Anaplasma phagocytophilum*: Phylogenetic tree of partial heat shock protein gene *groEL* of *Anaplasma phagocytophilum* of the four, one sequences is slightly different by couple of mismatches. All four are part of zoonotic variant of *Anaplasma phagocytophilum*. B.) *Babesia* genospecies: Three of the tested blood samples for *Babesia* genospecies yielded a sequence for the ribosomal *18S rRNA* gene, and showed to be identical to *B*. *divergens* sequences. C.) *Candidatus* Neoehrlichia mikurensis: Five out of seven *Candidatus* Neoehrlichia mikurensis yielded a partial sequence of the citrate synthase gene *gltA*. D.) *Candidatus* Neoehrlichia mikurensis: All seven *Candidatus* Neoehrlichia mikurensis yielded a partial sequence of the heat shock protein gene *groEL*.

All seven of the *Candidatus* Neoehrlichia mikurensis sequence yielded a partial *groEL* sequence and five out of seven could also be confirmed on a separate gene, namely *gltA*. The seven *groEL* are 100% identical to each other as were the five *gltA* sequences (Fig 1). Four out of five *A*. *phagocytophilum* positives yielded a partial *groEL* sequence after nested PCR. The four *groEL* are almost identical to each other, with just one or two mismatches. Nevertheless, all four sequences are part of zoonotic variant of the *A*. *phagocytophilum*, ecotype I [33]. Three of the tested blood samples for *Babesia* genospecies yielded a sequence in conventional PCR for the ribosomal *18S rRNA* gene, and showed to be identical to *B*. *divergens* sequences. Extensive efforts to generate a *B*. *miyamotoi* sequence failed. Accession numbers of the obtained sequences are: LC167302, LC167303, LC167304, LC167305.

### Characteristics of 16 participants with DNA of tick-borne pathogens detected in blood

The prevalence of DNA of a tick-borne pathogen other than *B*. *burgdorferi* s. l. detected in blood from persons after a tick bite was 2.4% (Table 2), this number was similar to EM patients (2.7%). In the blood of one person DNA of both *A*. *phagocytophilum* and *B*. *divergens* were detected. Altogether, DNA of tick-borne pathogens was detected in the blood of sixteen persons. The characteristics of these sixteen participants are summarized in Table 3, with regard to age, gender, self-reported clinical symptoms, antibiotic treatment and tick exposure at enrolment and during the three month follow-up period. Eight of the sixteen cases had submitted ticks at enrolment. Among these eight ticks, six ticks tested negative in PCR, and in two ticks DNA was detected of a different genus than the tick-borne pathogens that had been detected in the EDTA-blood of the corresponding participants (Table 3). We did not observe associations between detection of tick-borne pathogen DNA in EDTA-blood and; patient-reported tick attachment duration, with tick engorgement, with antibiotic treatment at enrolment. Seven out of sixteen cases reported clinical symptoms at enrolment or during the three month follow-up period, such as myalgia (3 cases), skin rash (2 cases), tingling sensations in limbs (2 cases), fatigue, arthralgia, headache, pain in limbs, and gastrointestinal symptoms/vomiting. Using multivariate logistic regression, we compared the prevalence of self-reported symptoms, tick attachment duration and tick engorgement among cases with and without DNA of tick-borne pathogens detected in blood. Compared to the cases that tested negative by PCR, the cases with DNA of tick-borne pathogens detected in their blood sample were not more likely to report any of the named clinical symptoms at enrolment or at follow-up.

**Table 3:** Characteristics of participants with DNA of tick-borne pathogens detected in blood.

Patient	EM and self-reported symptoms	Antibiotic treatment	Microorganism DNA detected in EDTA-blood	Tick (bite) characteristics: reported tick attachment duration, engorgement, detected DNA of microorganisms in tick	Reported tick exposure history
Case 1: Male, age 60	t = 0: EM	t = 0: doxycycline	*B*. *burgdorferi* s.l.	Reported attachment duration of tick bite before the EM at enrollment: 24 hours.	1 tick bite in past 7 days. No other tick bites in past 5 years.
		100 mg b.i.d. 10 days			
Case 2: Male, age 42	t = 0: EM	t = 0: doxycycline 100 mg b.i.d. 10 days	*B*. *miyamotoi*	Reported attachment duration of tick bite before the EM at enrollment: 72 hours.	1 tick bite in past 7 days. 3 other tick bites in past 5 years, >6 weeks ago.
Case 3: Female, age 58	t = 0: skin rash	No	*A*. *phagocytophilum*	Submitted tick: unengorged adult female *Ixodes ricinus*. Tick PCR positive for *B*. *burgdorferi* sensu stricto, Untypeable* Ehrlichia / Anaplasma spp, Ca. *Neoehrlichia mikurensis*, and *R*. *helvetica*. Reported tick attachment duration: 17 hours.	1 tick bite at t = 0. 30 other tick bites in past 5 years, 6 in past 6 weeks, 2 in past 7 days.
Case 4: Female, age 56	No	t = 12: doxycycline 1 wk treatment of bronchitis	*A*. *phagocytophilum*	Submitted tick: unengorged nymph *Ixodes ricinus*. Tick PCR negative. Reported tick attachment duration: 14 hours.	2 tick bites at t = 0. >15 other tick bites in past 5 years, 1 in past 6 weeks.
Case 5: Female, age 63	t = 0: myalgia, tingling in limbs, fatigue.	No	*A*. *phagocytophilum*	Submitted tick: unengorged nymph *Ixodes ricinus*. Tick PCR negative. Reported tick attachment duration: 2 hours.	1 tick bite at t = 0. No other tick bites in past 5 years.
	t = 12: vomiting, gastrointestinal symptoms.		*Babesia divergens*		
Case 6: Female, age 38	t = 0: EM	t = 0: amoxicillin	*A*. *phagocytophilum*	Submitted tick: partially engorged nymph *Ixodes ricinus*. Tick PCR negative. Reported tick attachment duration: 36 hours.	1 tick bite in past 10 days. 1 other tick bite in past 5 years, >6 weeks ago.
	t = 12: myalgia	500 mg q.i.d. 14 days			
Case 7: Female, age 46	t = 0: EM	t = 0: doxycycline 100 mg b.i.d. 14 days	*A*. *phagocytophilum*	Reported attachment duration of tick bite before the EM at enrollment: 25 hours.	2 tick bites in past 7 days. 2 other tick bites in past 5 years.
Case 8: Female, age 15	No	No	*Babesia divergens*	Submitted tick: partially engorged female adult *Ixodes ricinus*. Tick PCR negative. Reported tick attachment duration: 3 hours.	1 tick bite at t = 0. No other tick bites in past 5 years.
	No information on t = 12.				
Case 9: Male, age 55	t = 0: EM	t = 0: doxycycline 100 mg b.i.d. 14 days	*Babesia divergens*	Reported attachment duration of tick bite before the EM at enrollment: 96 hours.	2 tick bites in past 7 days. No other tick bites in past 5 years.
Case 10: Female, age 63	t = 12: arthralgia	No	*Ca*. Neoehrlichia mikurensis	Submitted tick: unengorged nymph *Ixodes ricinus*. Tick PCR positive for *R*. *helvetica*. Reported tick attachment duration: 8 hours.	1 tick bite at t = 0. 25 other tick bites in past 5 years, 4 in past 6 weeks.
Case 11: Male, age 79	No	No	*Ca*. Neoehrlichia mikurensis	Submitted tick: partially engorged female adult *Ixodes ricinus*. Tick PCR negative. Reported tick attachment duration: 12 hours.	1 tick bite at t = 0. No other tick bites in past 5 years.
Case 12: Male, age 40	t = 0: skin rash	t = 12: doxycycline	*Ca*. Neoehrlichia mikurensis	Reported attachment duration of tick bite before the EM at enrollment: 8 hours.	3 tick bites in past 3 weeks. Other tick bites (number unknown) in past 5 years, >6 weeks ago.
	t = 12: EM	100 mg b.i.d. 10 days			
Case 13: Female, age 60	t = 0: EM	t = 0: doxycycline	*Ca*. Neoehrlichia mikurensis	Unknown attachment duration of tick bite before the EM at enrollment.	1 tick bite in past 7 days. 10 other tick bites in past 5 years, 3 in past 6 weeks.
		100 mg b.i.d. 10 days			
Case 14: Female, age 61	t = 0: faded EM (not inspected by physician), headache, myalgia, pain in limbs.	t = 0: doxycycline 100 mg b.i.d. 14 days	*Ca*. Neoehrlichia mikurensis	Reported attachment duration of tick bite before the EM at enrollment: 16 hours.	1 tick bite in past weeks. No other tick bites in past 5 years.
	t = 12: myalgia.				
Case 15: Male, age 48	t = 0: EM, tingling in limbs.	t = 0: doxycycline	*Ca*. Neoehrlichia mikurensis	Reported attachment duration of tick bite before the EM at enrollment: 10 hours.	5 in past 7 days. >20 other tick bites in past 5 years, >6 weeks ago.
	No information on t = 12.	100 mg b.i.d. 10 days			
Case 16: Male, age 71	No	No	*Ca*. Neoehrlichia mikurensis	Submitted tick: partially engorged female adult *Ixodes ricinus*. Tick PCR negative. Unknown tick attachment duration.	1 tick bite at t = 0. No other tick bites in past 5 years.

EM: erythema migrans.

t = 0: time of enrolment, at the time of blood sample collection for PCR-testing.

t = 12: twelve weeks after enrolment.

b.i.d.: bis in die.

q.i.d.: quarter in die.

* PCR products from tick analyses that specifically reacted to the generic (“catch all”) probes, but that could not be further specified to the (geno)species level. were designated as 'Untypeable'.

## Discussion

In this study, DNA of tick-borne microorganisms was detected and identified in ticks and human blood samples (Tables 1 and 2). The limitations of this methodology are well known; hence, the interpretation of these results should be done with caution [34]. In order to unequivocally prove the presence of the corresponding infectious agents in ticks or blood, their viability should be tested by *in vitro* culture or infection experiments of laboratory animals. Also, the absence of DNA of a pathogen cannot be interpreted as the absence of the infectious agent. Besides the technical detection limits of PCR-based methods, the timing of sample collection after a tick bite and start of an antibiotic treatment, as well as the tissue tropism of the pathogen strongly affect the ability of pathogen detection [35, 36]. The latter is corroborated in this study: Only in one out of the 291 patients with an erythema migrans (EM) -a skin infection caused by *B*. *burgdorferi* s. l.—the DNA of this pathogen was detected in blood (Table 2). This finding confirms that the chance of detecting *B*. *burgdorferi* s. l. DNA in blood samples of confirmed Lyme borreliosis patients is very low [35]. *Rickettsia helvetica* and *R*. *monacensis* were both not detected in the 626 blood samples whereas, recently molecular evidence for their presence in cerebrospinal fluid of neuroborreliosis patients and in a skin sample of an EM patient was found [37, 38]. The absence of TBEV in blood samples can further be explained by its extremely low infection rates in ticks and focal geographic distribution in the Netherlands [15]. *Candidatus* Neoehrlichia mikurensis, *A*. *phagocytophilum*, *B*. *miyamotoi* and *Babesia* species are all pathogens that can be expected in blood because of their biology and tissue tropism [16, 20, 39, 40].

The tick samples were screened by a different method (RLB) than the blood samples (real-time PCR). In 314 ticks removed from humans a wide variety of tick-borne pathogens were detected namely, *Borrelia afzelii*, *Borrelia garinii*, *Borrelia burgdorferi* sensu stricto, *Borrelia valaisiana*, *Babesia microti*, *Babesia venatorum*, *Babesia divergens*, *Anaplasma phagocytophilum*, *Candidatus* Neoehrlichia mikurensis, *Rickettsia helvetica*, *Rickettsia monacensis* and *Borrelia miyamotoi*. All these pathogens have been found in questing ticks from field studies in the Netherlands before [41–43]. The infection rate of tick-borne pathogens other than *B*. *burgdorferi* s. l. varied from 0.3% (*B*. *divergens* and *R*. *monacensis*) up to 18.8*%* (*R*. *helvetica*). With an estimated incidence of 1.1 million tick bites per year, human exposure to a tick-borne pathogen other than *B*. *burgdorferi* s. l. and TBEV varies from roughly 3500 for *B*. *divergens*, and 3500 for *R*. *monacensis* to 207,000 persons for *R*. *helvetica*. Among the 322,000 persons exposed to *B*. *burgdorferi* s. l. through a tick bite, roughly 105,000 are simultaneously exposed to another pathogen. In addition, exposure to more than one tick-borne pathogen can occur when people have more than one tick bite at once or several consecutive tick bites.

Clearly, not all exposure to tick-borne pathogens results in human infection. Based on the development of an EM or seroconversion, the risk of infection with *B*. *burgdorferi* s. l. after tick bites was estimated to be 5.1% [29]. In this study, evidence for infection comes from the detection of *Candidatus* Neoehrlichia mikurensis, *A*. *phagocytophilum*, *B*. *divergens*, *B*. *miyamotoi* and *B*. *burgdorferi* s. l. DNA in the blood of sixteen individuals after exposure to a tick bite. None of these cases reported to be immunocompromised, and all the EM patients were treated with antibiotics according to the guidelines for treatment of Lyme borreliosis [30]. Mild clinical symptoms were reported by seven out of sixteen PCR-positive cases. However, using multivariate logistic regression, we did not detect associations between DNA detected in blood and self-reported symptoms at enrolment and follow-up. Furthermore, we did not find associations between detection of DNA of tick-borne pathogens in blood and; PCR positive ticks, patient-reported tick attachment duration, tick engorgement, and antibiotic treatment at enrolment. The lack of statistically significant associations may be due to the mildness of symptoms amongst immune-competent patients, and to a lesser degree due to insufficient numbers of PCR-positive cases per pathogen genus in our analyses.

In this study, *Candidatus* Neoehrlichia mikurensis infection was observed in 1.1% (95%CI 0.5%–2.2%). *Candidatus* Neoehrlichia mikurensis infections have been described in immunocompromised patients [44], and more recently in immune-competent patients with relatively mild symptoms in China, Poland, and Sweden [45–48]. *Anaplasma phagocytophilum* infection was found in 0.9% (95%CI 0.3%–2.0%) of the persons exposed to tick bites in the Netherlands (Table 2). Genetic analyses of the DNA sequences showed the highest similarity to the zoonotic *A*. *phagocytophilum* ecotype I [33]. Evidence for *A*. *phagocytophilum* infection is primarily based on its molecular, microscopic or serological detection most disease cases [20, 49]. There is serological evidence that *A*. *phagocytophilum* infection occurs in the absence of disease symptoms [50]. *Babesia divergens* infection was observed in 0.5% (95%CI 0.1%–1.3%) of the persons exposed to tick bites. In Europe, only two cases of human babesiosis have been reported in immune-competent patients, one due to *B*. *divergens* [51]. Only one patient with EM was possibly infected with *B*. *miyamotoi* 0.2% (95%CI 0.0%–0.8%). The presence of *B*. *miyamotoi* DNA could only be determined by real-time PCR, and several attempts to confirm this finding by conventional PCR was unsuccessful. This patient had received antibiotic treatment at enrolment for his EM, so a low bacterial load in blood due to the treatment could be an explanation for the high Ct value. Evidence for infection with *B*. *miyamotoi* in Europe comes from one immunocompromised case [18], and a seroprevalence study in people exposed to tick bites [52].

Altogether, the probability of infection with a tick-borne pathogen other than Lyme spirochetes after tick bites in the Netherlands is roughly 2.4% (95%CI 1.1%–4.5%). This number is similar to the probability of a co-infection with another tick-borne pathogen in patients with EM (2.7%, 95%CI 1.3%–5.2%). Interestingly, one patient in this study had a co-infection with *A*. *phagocytophilum* and *B*. *divergens*. The severity of self-reported symptoms of the seven EM patients with a co-infection was indistinguishable from patients only having EM. No indications were found that infection with a tick-borne pathogen other than *B*. *burgdorferi* s. l. caused overt symptoms that would indicate a corresponding illness. The low number of persons with a tick bite or EM that were identified with an tick-borne pathogen infection other than *B*. *burgdorferi* s. l., in combination with the limited medical assessments, and the used method of pathogen detection are not sufficient to infer how often tick-borne pathogens other than *B*. *burgdorferi* s. l. (and TBEV) cause disease. Also, to what extend they affect the diagnoses and the etiology of Lyme borreliosis. Furthermore, the ability for a pathogen to cause disease depends also on extrinsic factors for example the immune status of its host.

The high exposure to tick-borne pathogens other than *B*. *burgdorferi* s. l. and TBEV, and their ability to cause infection in the general population, warrants increased awareness, knowledge, improvement of diagnostic tests and a clear-cut clinical case definitions in an European setting. Only when better laboratory tests are available for these tick-borne diseases, their impact as a co-infection with Lyme borreliosis can be assessed.

## Supporting Information

S1 Checklist STROBE Statement-Checklist for the cohort study. (DOC)

S1 Table Primers and probes used in this study for the real-time PCRs. (DOCX)

S2 Table Primers used in this study for conventional PCR. (DOCX)

## References

[1] Stanek G, Fingerle V, Hunfeld KP, Jaulhac B, Kaiser R, Krause A, et al Lyme borreliosis: clinical case definitions for diagnosis and management in Europe. Clinical Microbiology and Infection. 2011;17(1):69–79. 10.1111/j.1469-0691.2010.03175.x 20132258

[2] Stanek G, Reiter M. The expanding Lyme Borrelia complex—clinical significance of genomic species? Clinical Microbiology and Infection. 2011;17(4):487–93. 10.1111/j.1469-0691.2011.03492.x 21414082

[3] Stanek G, Wormser GP, Gray J, Strle F. Lyme borreliosis. The Lancet. 2012;379(9814):461–73. 10.1016/S0140-6736(11)60103-7 21903253

[4] Bogovic P, Strle F. Tick-borne encephalitis: A review of epidemiology, clinical characteristics, and management. World J Clin Cases. 2015;3(5):430–41. 10.12998/wjcc.v3.i5.430 25984517 PMC4419106

[5] Hubálek Z. Epidemiology of Lyme borreliosis. 2009. 10.1159/000213069 19367096

[6] Smith R, Takkinen J. Lyme borreliosis: Europe-wide coordinated surveillance and action needed. Euro Surveill. 2006;11(6):E060622 16819127 10.2807/esw.11.25.02977-en

[7] Lindgren E, Andersson Y, Suk JE, Sudre B, Semenza JC. Public health. Monitoring EU emerging infectious disease risk due to climate change. Science. 2012;336(6080):418–9. 10.1126/science.1215735 . 22539705

[8] Hofhuis A, Harms M, van den Wijngaard C, Sprong H, van Pelt W. Continuing increase of tick bites and Lyme disease between 1994 and 2009. Ticks and tick-borne diseases. 2015;6(1):69–74. 10.1016/j.ttbdis.2014.09.006 . 25448421

[9] de Mik EL, van Pelt W, Docters-van Leeuwen B, van der Veen A, Schellekens J, Borgdorff MW. The geographical distribution of tick bites and erythema migrans in general practice in The Netherlands. International journal of epidemiology. 1997;26(2):451–7. 9169184 10.1093/ije/26.2.451

[10] Wielinga PR, Fonville M, Sprong H, Gaasenbeek C, Borgsteede F, van der Giessen JW. Persistent detection of Babesia EU1 and Babesia microti in Ixodes ricinus in the Netherlands during a 5-year surveillance: 2003–2007. Vector-Borne and Zoonotic Diseases. 2009;9(1):119–22. 10.1089/vbz.2008.0047 . 18759637

[11] Wielinga PR, Gaasenbeek C, Fonville M, de Boer A, de Vries A, Dimmers W, et al Longitudinal analysis of tick densities and Borrelia, Anaplasma, and Ehrlichia infections of Ixodes ricinus ticks in different habitat areas in The Netherlands. Applied and environmental microbiology. 2006;72(12):7594–601. 10.1128/AEM.01851-06 17028227 PMC1694262

[12] Coipan EC, Jahfari S, Fonville M, Maassen CB, van der Giessen J, Takken W, et al Spatiotemporal dynamics of emerging pathogens in questing Ixodes ricinus. Front Cell Infect Microbiol. 2013;3:36 10.3389/fcimb.2013.00036 23908971 PMC3726834

[13] Jahfari S, Fonville M, Hengeveld P, Reusken C, Scholte EJ, Takken W, et al Prevalence of Neoehrlichia mikurensis in ticks and rodents from North-west Europe. Parasites & vectors. 2012;5:74 10.1186/1756-3305-5-74 22515314 PMC3395572

[14] Sprong H, Wielinga PR, Fonville M, Reusken C, Brandenburg AH, Borgsteede F, et al Ixodes ricinus ticks are reservoir hosts for Rickettsia helvetica and potentially carry flea-borne Rickettsia species. Parasites & vectors. 2009;2(1):41 10.1186/1756-3305-2-41 19732416 PMC2743653

[15] Jahfari S, de Vries, A., Rijks, J., van Gucht, S., Sprong, H., Rockx, B. Tick-borne encephalitis virus in ticks and roe deer, the Netherlands. submitted. 2016. 10.3201/eid2306.161247 PMC5443429 28518024

[16] Wagemakers A, Staarink PJ, Sprong H, Hovius JW. Borrelia miyamotoi: a widespread tick-borne relapsing fever spirochete. Trends in parasitology. 2015;31(6):260–9. 10.1016/j.pt.2015.03.008 . 25892254

[17] Grankvist A, Andersson P-O, Mattsson M, Sender M, Vaht K, Höper L, et al Infections with the tick-borne bacterium “Candidatus Neoehrlichia mikurensis” mimic noninfectious conditions in patients with B cell malignancies or autoimmune diseases. Clinical Infectious Diseases. 2014;58(12):1716–22. 10.1093/cid/ciu189 24647019

[18] Hovius JW, de Wever B, Sohne M, Brouwer MC, Coumou J, Wagemakers A, et al A case of meningoencephalitis by the relapsing fever spirochaete Borrelia miyamotoi in Europe. The Lancet. 2013;382(9892):658 10.1016/S0140-6736(13)61644-X 23953389 PMC3987849

[19] Rajkumari N. Epidemiological profile of "Babesia venatorum". Lancet Infect Dis. 2015;15(8):877–8. 10.1016/S1473-3099(15)00141-3 . 26227757

[20] Stuen S, Granquist EG, Silaghi C. Anaplasma phagocytophilum—a widespread multi-host pathogen with highly adaptive strategies. Front Cell Infect Microbiol. 2013;3:31 10.3389/fcimb.2013.00031 23885337 PMC3717505

[21] Hunfeld K-P, Hildebrandt A, Gray J. Babesiosis: recent insights into an ancient disease. International journal for parasitology. 2008;38(11):1219–37. 10.1016/j.ijpara.2008.03.001 18440005

[22] Andréasson K, Jönsson G, Lindell P, Gülfe A, Ingvarsson R, Lindqvist E, et al Recurrent fever caused by Candidatus Neoehrlichia mikurensis in a rheumatoid arthritis patient treated with rituximab. Rheumatology. 2015;54(2):369–71. 10.1093/rheumatology/keu441 25416710

[23] van Dobbenburgh A, van Dam AP, Fikrig E. Human granulocytic ehrlichiosis in western Europe. New England Journal of Medicine. 1999;340(15):1214–6. 10.1056/NEJM199904153401516 10206853

[24] Belongia EA. Epidemiology and impact of coinfections acquired from Ixodes ticks. Vector-Borne and Zoonotic Diseases. 2002;2(4):265–73. 10.1089/153036602321653851 12804168

[25] Swanson SJ, Neitzel D, Reed KD, Belongia EA. Coinfections acquired from Ixodes ticks. Clinical Microbiology Reviews. 2006;19(4):708–27. 10.1128/CMR.00011-06 17041141 PMC1592693

[26] Krause PJ, Foley DT, Burke GS, Christianson D, Closter L, Spielman A, et al Reinfection and relapse in early Lyme disease. The American journal of tropical medicine and hygiene. 2006;75(6):1090–4. 17172372

[27] Wormser GP, Dattwyler RJ, Shapiro ED, Halperin JJ, Steere AC, Klempner MS, et al The clinical assessment, treatment, and prevention of lyme disease, human granulocytic anaplasmosis, and babesiosis: clinical practice guidelines by the Infectious Diseases Society of America. Clinical Infectious Diseases. 2006;43(9):1089–134. 10.1086/508667 . 17029130

[28] Krause PJ, Telford SR, Spielman A, Sikand V, Ryan R, Christianson D, et al Concurrent Lyme disease and babesiosis: evidence for increased severity and duration of illness. Jama. 1996;275(21):1657–60. 8637139

[29] Hofhuis A, Herremans T, Notermans DW, Sprong H, Fonville M, van der Giessen JW, et al A prospective study among patients presenting at the general practitioner with a tick bite or erythema migrans in The Netherlands. PLoS One. 2013;8(5):e64361 10.1371/journal.pone.0064361 23696884 PMC3655959

[30] Speelman P, De Jongh B, Wolfs T, Wittenberg J. [Guideline'Lyme borreliosis']. Nederlands tijdschrift voor geneeskunde. 2004;148(14):659–63. 15106316

[31] Tijsse-Klasen E, Fonville M, Reimerink JH, Spitzen-van der Sluijs A, Sprong H. Role of sand lizards in the ecology of Lyme and other tick-borne diseases in the Netherlands. Parasites & vectors. 2010;3:42 10.1186/1756-3305-3-42 20470386 PMC2890652

[32] Lindblom P, Wilhelmsson P, Fryland L, Sjöwall J, Haglund M, Matussek A, et al Tick-borne encephalitis virus in ticks detached from humans and follow-up of serological and clinical response. Ticks and tick-borne diseases. 2014;5(1):21–8. 10.1016/j.ttbdis.2013.07.009 24275477

[33] Jahfari S, Coipan EC, Fonville M, van Leeuwen AD, Hengeveld P, Heylen D, et al Circulation of four Anaplasma phagocytophilum ecotypes in Europe. Parasites & vectors. 2014;7:365 10.1186/1756-3305-7-365 25127547 PMC4153903

[34] Tijsse-Klasen E, Koopmans MP, Sprong H. Tick-borne pathogen—reversed and conventional discovery of disease. Front Public Health. 2014;2:73 10.3389/fpubh.2014.00073 25072045 PMC4083466

[35] Cerar T, Ogrinc K, Cimperman J, Lotrič-Furlan S, Strle F, Ružić-Sabljić E. Validation of cultivation and PCR methods for diagnosis of Lyme neuroborreliosis. Journal of clinical microbiology. 2008;46(10):3375–9. 10.1128/JCM.00410-08 18716226 PMC2566093

[36] Elfving K, Lukinius A, Nilsson K. Life cycle, growth characteristics and host cell response of Rickettsia helvetica in a Vero cell line. Experimental and applied acarology. 2012;56(2):179–87. 10.1007/s10493-011-9508-7 22116301 PMC3253991

[37] Tijsse-Klasen E, Sprong H, Pandak N. Co-infection of Borrelia burgdorferi sensu lato and Rickettsia species in ticks and in an erythema migrans patient. Parasites & vectors. 2013;6:347 10.1186/1756-3305-6-347 24326096 PMC3878868

[38] Koetsveld J, Tijsse-Klasen E, Herremans T, Hovius JW, Sprong H. Serological and molecular evidence for spotted fever group Rickettsia and Borrelia burgdorferi sensu lato co-infections in The Netherlands. Ticks and tick-borne diseases. 2015 10.1016/j.ttbdis.2015.12.010 . 26739030

[39] Silaghi C, Beck R, Oteo JA, Pfeffer M, Sprong H. Neoehrlichiosis: an emerging tick-borne zoonosis caused by Candidatus Neoehrlichia mikurensis. Experimental and Applied Acarology. 2016;68(3):279–97. 10.1007/s10493-015-9935-y 26081117

[40] Hildebrandt A, Gray J, Hunfeld K-P. Human babesiosis in Europe: what clinicians need to know. Infection. 2013;41(6):1057–72. 10.1007/s15010-013-0526-8 24104943

[41] Coipan EC, Fonville M, Tijsse-Klasen E, van der Giessen JW, Takken W, Sprong H, et al Geodemographic analysis of Borrelia burgdorferi sensu lato using the 5S-23S rDNA spacer region. Infect Genet Evol. 2013;17:216–22. 10.1016/j.meegid.2013.04.009 . 23602839

[42] Cochez C, Heyman P, Heylen D, Fonville M, Hengeveld P, Takken W, et al The Presence of Borrelia miyamotoi, A Relapsing Fever Spirochaete, in Questing Ixodes ricinus in Belgium and in The Netherlands. Zoonoses Public Health. 2015;62(5):331–3. 10.1111/zph.12154 . 25212814

[43] Jacobs JJ, Noordhoek GT, Brouwers JM, Wielinga PR, Jacobs JP, Brandenburg AH. [Small risk of developing Lyme borreliosis following a tick bite on Ameland: research in a general practice]. Ned Tijdschr Geneeskd. 2008;152(37):2022–6. . 18825891

[44] Silaghi C, Beck R, Oteo JA, Pfeffer M, Sprong H. Neoehrlichiosis: an emerging tick-borne zoonosis caused by Candidatus Neoehrlichia mikurensis. Experimental and applied acarology. 2016;68(3):279–97. 10.1007/s10493-015-9935-y . 26081117

[45] Welc-Falęciak R, Siński E, Kowalec M, Zajkowska J, Pancewicz SA. Asymptomatic “Candidatus Neoehrlichia mikurensis” infections in immunocompetent humans. Journal of clinical microbiology. 2014;52(8):3072–4. 10.1128/JCM.00741-14 24899023 PMC4136151

[46] Li H, Jiang J-F, Liu W, Zheng Y-C, Huo Q-B, Tang K, et al Human infection with Candidatus Neoehrlichia mikurensis, China. Emerging Infectious Diseases. 2012;18(10):1636–9. 10.3201/eid1810.120594 23017728 PMC3471638

[47] Grankvist A, Moore ER, Stadler LS, Pekova S, Bogdan C, Geißdörfer W, et al Multilocus Sequence Analysis of Clinical “Candidatus Neoehrlichia mikurensis” Strains from Europe. Journal of clinical microbiology. 2015;53(10):3126–32. 10.1128/JCM.00880-15 26157152 PMC4572549

[48] Grankvist A, Sandelin LL, Andersson J, Fryland L, Wilhelmsson P, Lindgren P-E, et al Infections with Candidatus neoehrlichia mikurensis and cytokine responses in 2 persons bitten by ticks, Sweden. Emerging infectious diseases. 2015;21(8):1462 10.3201/eid2108.150060 26197035 PMC4517700

[49] Rizzoli A, Silaghi C, Obiegala A, Rudolf I, Hubalek Z, Foldvari G, et al Ixodes ricinus and Its Transmitted Pathogens in Urban and Peri-Urban Areas in Europe: New Hazards and Relevance for Public Health. Front Public Health. 2014;2:251 10.3389/fpubh.2014.00251 25520947 PMC4248671

[50] Henningsson AJ, Wilhelmsson P, Gyllemark P, Kozak M, Matussek A, Nyman D, et al Low risk of seroconversion or clinical disease in humans after a bite by an Anaplasma phagocytophilum-infected tick. Ticks and tick-borne diseases. 2015;6(6):787–92. 10.1016/j.ttbdis.2015.07.005 26187418

[51] Martinot M, Zadeh MM, Hansmann Y, Grawey I, Christmann D, Aguillon S, et al Babesiosis in immunocompetent patients, Europe. Emerging Infectious Diseases. 2011;17(1):114–6. 10.3201/eid1701.100737 21192869 PMC3204631

[52] Jahfari S, Herremans T, Platonov AE, Kuiper H, Karan LS, Vasilieva O, et al High seroprevalence of Borrelia miyamotoi antibodies in forestry workers and individuals suspected of human granulocytic anaplasmosis in the Netherlands. New Microbes and New Infections. 2014;2(5):144–9. 10.1002/nmi2.59 25356364 PMC4184479

