# Supplementary material for: Molecular Detection of Tick-Borne Pathogens in Humans with Tick Bites and Erythema Migrans, in the Netherlands
Source: PLoS Negl Trop Dis. 2016 Oct 5;10(10):e0005042. doi: 10.1371/journal.pntd.0005042 (PMC5051699; doi:10.1371/journal.pntd.0005042)
Supplement: S1 Checklist — (DOC) [file pntd.0005042.s001.doc]

STROBE Statement—Checklist of items that should be included in reports of ***cohort studies***

|  | Item No | Recommendation | Check for PNTD-D-16-01245R1 |  | |
| --- | --- | --- | --- | --- | --- |
| **Title and abstract** | 1 | (*a*) Indicate the study’s design with a commonly used term in the title or the abstract | Cohort study |  | |
|  |  | (*b*) Provide in the abstract an informative and balanced summary of what was done and what was found | ✓ Manuscript abstract & author summary |  | |
| Introduction | | |  |  |  |
| Background/ rationale | 2 | Explain the scientific background and rationale for the investigation being reported | ✓ Manuscript introduction |  | |
| Objectives | 3 | State specific objectives, including any prespecified hypotheses | ✓ Manuscript page 5 (end of introduction) |  | |
| Methods | | |  |  | |
| Study design | 4 | Present key elements of study design early in the paper | ✓ Manuscript page 5 (end of introduction),  and page 6,7,8 (Methods) |  | |
| Setting | 5 | Describe the setting, locations, and relevant dates, including periods of recruitment, exposure, follow-up, and data collection | ✓ Manuscript methods, “Study design, ticks, human samples and questionnaires”, and referral to publication Hofhuis A. *et al*. A prospective study among patients presenting at the general practitioner with a tick bite or erythema migrans in The Netherlands. PLoS One. 2013; 8(5): e64361. |  | |
| Participants | 6 | (*a*) Give the eligibility criteria, and the sources and methods of selection of participants. Describe methods of follow-up | ✓ Manuscript methods, “Study design, ticks, human samples and questionnaires”, and referral to publication Hofhuis 2013. |  | |
|  |  | (*b*) For matched studies, give matching criteria and number of exposed and unexposed | ✓ Not applicable |  | |
| Variables | 7 | Clearly define all outcomes, exposures, predictors, potential confounders, and effect modifiers. Give diagnostic criteria, if applicable | ✓ Manuscript methods, “Statistical analyses”. |  | |
| Data sources/ measurement | 8* | For each variable of interest, give sources of data and details of methods of assessment (measurement). Describe comparability of assessment methods if there is more than one group | ✓ Manuscript methods. |  | |
| Bias | 9 | Describe any efforts to address potential sources of bias | ✓ Manuscript discussion, e.g.:  “the absence of DNA of a pathogen cannot be interpreted as the absence of the infectious agent”  &  “The lack of statistically significant associations may be due to the mildness of symptoms amongst immune-competent patients, and to a lesser degree due to insufficient numbers of PCR-positive cases per pathogen genus in our analyses.” |  | |
| Study size | 10 | Explain how the study size was arrived at | ✓ Manuscript page 6, and referral to publication Hofhuis 2013. |  | |
| Quantitative variables | 11 | Explain how quantitative variables were handled in the analyses. If applicable, describe which groupings were chosen and why | ✓ Manuscript methods, “Statistical analyses”. |  | |
| Statistical methods | 12 | (*a*) Describe all statistical methods, including those used to control for confounding | ✓ Manuscript methods, “Statistical analyses”. |  | |
|  |  | (*b*) Describe any methods used to examine subgroups and interactions | ✓ Manuscript methods, “Statistical analyses”. |  | |
|  |  | (*c*) Explain how missing data were addressed | ✓ Manuscript methods, “Statistical analyses”, and referral to publication Hofhuis 2013. |  | |
|  |  | (*d*) If applicable, explain how loss to follow-up was addressed | ✓ Manuscript methods, “Statistical analyses”, and referral to publication Hofhuis 2013. |  | |
|  |  | (*e*) Describe any sensitivity analyses | ✓ Not applicable |  | |
| Results | | |  |  | |
| Participants | 13* | (a) Report numbers of individuals at each stage of study—eg numbers potentially eligible, examined for eligibility, confirmed eligible, included in the study, completing follow-up, and analysed | Referral to publication Hofhuis 2013. |  | |
|  |  | (b) Give reasons for non-participation at each stage | Referral to publication Hofhuis 2013. |  | |
|  |  | (c) Consider use of a flow diagram | Referral to publication Hofhuis 2013. |  | |
| Descriptive data | 14* | (a) Give characteristics of study participants (eg demographic, clinical, social) and information on exposures and potential confounders | For persons with infection:  Table 3. Characteristics of participants with DNA of tick-borne pathogens detected in blood.  For the total group of subjects:  ✓ Manuscript methods, “Study design, ticks, human samples and questionnaires”, and referral to publication Hofhuis 2013. |  | |
|  |  | (b) Indicate number of participants with missing data for each variable of interest | For persons with infection:  Table 3. Characteristics of participants with DNA of tick-borne pathogens detected in blood.  For the total group of subjects:  ✓ Manuscript methods, “Study design, ticks, human samples and questionnaires”, and referral to publication Hofhuis 2013. |  | |
|  |  | (c) Summarise follow-up time (eg, average and total amount) | ✓ Manuscript methods, “Study design, ticks, human samples and questionnaires”, and referral to publication Hofhuis 2013. |  | |
| Outcome data | 15* | Report numbers of outcome events or summary measures over time | ✓ Manuscript results & tables. |  | |
| Main results | 16 | (*a*) Give unadjusted estimates and, if applicable, confounder-adjusted estimates and their precision (eg, 95% confidence interval). Make clear which confounders were adjusted for and why they were included | ✓ Manuscript results Table 1 & 2. |  | |
|  |  | (*b*) Report category boundaries when continuous variables were categorized | Referral to publication Hofhuis 2013. |  | |
|  |  | (*c*) If relevant, consider translating estimates of relative risk into absolute risk for a meaningful time period | ✓ Manuscript results Table 1 & 2. |  | |
| Other analyses | 17 | Report other analyses done—eg analyses of subgroups and interactions, and sensitivity analyses | ✓ Manuscript results Table 1 & 2: Estimation of human exposure with 1.1 million tick bites per year. |  | |
| Discussion | | |  |  | |
| Key results | 18 | Summarise key results with reference to study objectives | ✓ Manuscript discussion, 1^st^ sentence:  “In this study, DNA of tick-borne microorganisms was detected and identified in ticks and human blood samples (Table 1 and 2).” |  | |
| Limitations | 19 | Discuss limitations of the study, taking into account sources of potential bias or imprecision. Discuss both direction and magnitude of any potential bias | ✓ Manuscript discussion, e.g 2^nd^ sentence:  “The limitations of this methodology are well known; hence, the interpretation of these results should be done with caution...”  &  “Clearly, not all exposure to tick-borne pathogens results in human infection...” |  | |
| Interpretation | 20 | Give a cautious overall interpretation of results considering objectives, limitations, multiplicity of analyses, results from similar studies, and other relevant evidence | ✓ Manuscript discussion, e.g: “Altogether, the probability of infection with a tick-borne pathogen other than Lyme spirochetes after tick bites in the Netherlands is roughly 2.4% (95%CI 1.1% – 4.5%).” |  | |
| Generalisability | 21 | Discuss the generalisability (external validity) of the study results | ✓ Manuscript discussion, e.g: “The high exposure to tick-borne pathogens other than B. burgdorferi s. l. and TBEV, and their ability to cause infection in the general population, warrants increased awareness, knowledge, improvement of diagnostic tests and a clear-cut clinical case definitions in an European setting. Only when better laboratory tests are available for these tick-borne diseases, their impact as a co-infection with Lyme borreliosis can be assessed.” |  | |
| Other information | | |  |  | |
| Funding | 22 | Give the source of funding and the role of the funders for the present study and, if applicable, for the original study on which the present article is based | ✓ This study was financed by, and conducted on behalf of, the ministry of Health, Welfare and Sport of the Netherlands. The funders had no role in study design, data collection and analysis, decision to publish, or preparation of the manuscript. |  | |

*Give information separately for exposed and unexposed groups.

**Note:** An Explanation and Elaboration article discusses each checklist item and gives methodological background and published examples of transparent reporting. The STROBE checklist is best used in conjunction with this article (freely available on the Web sites of PLoS Medicine at http://www.plosmedicine.org/, Annals of Internal Medicine at http://www.annals.org/, and Epidemiology at http://www.epidem.com/). Information on the STROBE Initiative is available at http://www.strobe-statement.org.
