# Supplementary material for: Molecular Detection of Tick-Borne Pathogens in Humans with Tick Bites and Erythema Migrans, in the Netherlands
Source: PLoS Negl Trop Dis. 2016 Oct 5;10(10):e0005042. doi: 10.1371/journal.pntd.0005042 (PMC5051699; doi:10.1371/journal.pntd.0005042)
Supplement: S1 Table — (DOCX) [file pntd.0005042.s002.docx]

**Supplementary Table 1.**

| Microorganism | Target gene | Name | Sequence (sense) | Reference |
| --- | --- | --- | --- | --- |
| *Anaplasma phagocytophilum* | *msp2* | ApMSP2F | 5'-ATGGAAGGTAGTGTTGGTTATGGTATT-3' | [1] |
|  |  | ApMSP2R | 5'-TTGGTCTTGAAGCGCTCGTA-3' |  |
|  |  | ApMSP2P | 5'-FAM530-TGGTGCCAGGGTTGAGCTTGAGATTG-BHQ1-3' |  |
| *Candidatus* Neoehrlichia mikurensis | *groEL* | GroEL-F2a | 5'-CCTTGAAAATATAGCAAGATCAGGTAG-3' | [2] |
|  |  | GroEL-R2a | 5'-CCACCACGTAACTTATTTAGCACTAAAG-3' |  |
|  |  | GroEL-P2a | 5'-X-CCTCTACTAATTATTGCtGAAGATGTAGAAGGTGAAGC-BHQ2-3' X= CALFluorRed590nm |  |
| *Borrelia burgdorferi* s. l. | *ospA* | B-OspA_modF | 5'-AAT ATT TAT TGG GAA TAG GTC TAA-3' | [3] |
|  |  | B-OspA_borAS | 5'-CTTTGTCTTTTTCTTTRCTTACA-3' |  |
|  |  | B-OspAmodPatto | 5'-Atto520-AAG CAA AATGTTAGC AGC CTT GA-BHQ1-3' |  |
|  | *flaB* | B-FlaB-F | 5'-CAGAIAGAGGTTCTATACAIATTGAIATAGA-3' |  |
|  |  | B-FlaB-Rc | 5'-GTGCATTTGGTTAIATTGCGC-3' |  |
|  |  | B-FlaB-Rt | 5'-GTGCATTTGGTTAIATTGTGC-3' |  |
|  |  | B-FlaB-Patto | 5'-Atto425-CAACTIACAGAIGAAAXTAAIAGAATTGCTGAI CA-Pho-3' X = BHQ-1-dT |  |
| *Borrelia miyamotoi* | *flaB* | FlabBm.motoiF2 | 5'-AGAAGGTGCTCAAGCAG-3' | [4] |
|  |  | FlabB.m.motoiR3 | 5'-TCGATCTTTGAAAGTGACATA T-3' |  |
|  |  | FlabBm.motoiPro | 5'-ATTO647N-AGCACAACAGGAGGGAGTTCAAGC-BHQ2-3' |  |
| *Rickettsia* genospecies | *gltA* | RickgltA-F-Stenos | 5'- TCGCAAATGTTCACGGTACTTT -3' | [5] |
|  |  | RickgltA-R-Stenos | 5'- TCGTGCATTTCTTTCCATTGTG -3' |  |
|  |  | Rickglt-probe-stenos | 5'- Atto520-TGCAATAGCAAGAAC CGTAGG CTGGATG-BHQ1 -3' |  |
| *Rickettsia helvetica* | *gltA* | Rick_HelvgltA_F2 | 5'- ATGATCCGTTTAGGTTAATAGGCTTCGGTC -3' | [6] |
|  |  | Rick_HelvgltA_R2 | 5'- TTGTAAGAGCGGATTGTTTTCTAGCTGTC -3' |  |
|  |  | Rick_HelvgltA_pr3 | 5'-Atto425-CGATC+C+ACG+TG+CCGCAGT-BHQ1-3'  X = BHQ-1-dT |  |
| Tick-borne encephalitis virus | 3′ non-coding region | F-TBE | 5'-GGGCGGTTCTTGTTCTCC-3' | [7] |
|  |  | R-TBE | 5'-ACACATCACCTCCTTGTCAGACT-3' |  |
|  |  | TBE-Probe-WT | 5'-FAM-TGAGCCACCATCACCCAGACACA-BHQ1-3' |  |
|  | *E* gene | TBEE-F6 | 5'-GGCTTGTGAGGCAAAAAAGAA-3' | [8] |
|  |  |  | 5'-TCCCGTGTGTGGTTCGACTT-3' |  |
|  |  |  | 5'-JOE-AAGCCACAGGACATGTGTACGACGCC-BHQ2-3' |  |

1. Courtney JW, Kostelnik LM, Zeidner NS, Massung RF. Multiplex real-time PCR for detection of Anaplasma phagocytophilum and Borrelia burgdorferi. Journal of Clinical Microbiology. 2004;42(7):3164-8.

2. Jahfari S, Fonville M, Hengeveld P, Reusken C, Scholte EJ, Takken W, et al. Prevalence of Neoehrlichia mikurensis in ticks and rodents from North-west Europe. Parasites & vectors. 2012;5:74. doi: 10.1186/1756-3305-5-74. PubMed PMID: 22515314; PubMed Central PMCID: PMCPMC3395572.

3. Heylen D, Matthysen E, Fonville M, Sprong H. Songbirds as general transmitters but selective amplifiers of Borrelia burgdorferi sensu lato genotypes in Ixodes rinicus ticks. Environmental microbiology. 2014;16(9):2859-68. doi: 10.1111/1462-2920.12304. PubMed PMID: 24118930.

4. Hovius JW, de Wever B, Sohne M, Brouwer MC, Coumou J, Wagemakers A, et al. A case of meningoencephalitis by the relapsing fever spirochaete Borrelia miyamotoi in Europe. The Lancet. 2013;382(9892):658. doi: 10.1016/S0140-6736(13)61644-X. PubMed PMID: 23953389; PubMed Central PMCID: PMCPMC3987849.

5. Stenos J, Graves SR, Unsworth NB. A highly sensitive and specific real-time PCR assay for the detection of spotted fever and typhus group rickettsiae. The American journal of tropical medicine and hygiene. 2005;73(6):1083-5.

6. Heylen D, Fonville M, Leeuwen AD, Sprong H. Co‐infections and transmission dynamics in a tick‐borne bacterium community exposed to songbirds. Environmental microbiology. 2016.

7. Schwaiger M, Cassinotti P. Development of a quantitative real-time RT-PCR assay with internal control for the laboratory detection of tick borne encephalitis virus (TBEV) RNA. Journal of Clinical Virology. 2003;27(2):136-45.

8. Gäumann R, Mühlemann K, Strasser M, Beuret CM. High-throughput procedure for tick surveys of tick-borne encephalitis virus and its application in a national surveillance study in Switzerland. Applied and environmental microbiology. 2010;76(13):4241-9.
