# Supplementary material for: Molecular Detection of Tick-Borne Pathogens in Humans with Tick Bites and Erythema Migrans, in the Netherlands
Source: PLoS Negl Trop Dis. 2016 Oct 5;10(10):e0005042. doi: 10.1371/journal.pntd.0005042 (PMC5051699; doi:10.1371/journal.pntd.0005042)
Supplement: S2 Table — (DOCX) [file pntd.0005042.s003.docx]

**S2 Table 2.**

| Microorganism | Target gene | Name | Sequence (sense) |
| --- | --- | --- | --- |
| *Anaplasma phagocytophilum* | *groEL* | Ap-groEL-For | 5'-ATGGTATGCAGTTTGATCGC-3' |
|  |  | Ap-groEL-Rev | 5'-TTGAGTACAGCAACACCACCGGAA-3' |
|  |  | Ap-groEL_nested_For | 5'-GTGGAATTTGAAAATCCATAC-3' |
|  |  | Ap-groEL_nested_Rev | 5'-GTCCTGCTAGCTATGCTTTC-3' |
| *Candidatus* Neoehrlichia mikurensis | *groEL* | Neoehrl-groEL-For | 5'-GAAGCATAGTCTAGTATTTTTGTC-3' |
|  |  | Neoehrl-groEL-Rev | 5'-TTAACTTCTACTTCACTTG-3' |
|  |  | Neoehrl-groEL_nested-1 | 5'-ACATCACGCTTCATAGAA-3' |
|  |  | Neoehrl-groEL_nesetd-2 | 5'-AAAGGAATTAGTATTAGAATCTTT-3' |
|  |  | Neoehrl-groEL_nested-3 | 5'-AATATAGCAAGATCAGGTAGAC-3' |
|  |  | Neoehrl-groEL_nested-4 | 5'-CTTCCATTTTAACTGCTAA-3' |
| *Candidatus* Neoehrlichia mikurensis | *gltA* | Neoerhl-gltA-For | 5'-AAGTGCATGCTTTGCTACATT-3' |
|  |  | Neoerhl-gltA-Rev | 5'-TCATGATCTGCATGAAAATAA AT-3' |
| *Borrelia miyamotoi* | *glpQ* | glpQ-BM-F1 | 5'-CTCATAATTTCATGCTTTAAACAAGAAATG-3' |
|  |  | glpQ-BM-F2 | 5'-ATGGGTTCAAACAAAAAGTCACC-3' |
|  |  | glpQ-BM-F3 | 5'-GCTCACAGGGGTGCTAGTGGG-3' |
|  |  | glpQ-BM-R1 | 5'-CCAGGGTCCAATTCCATCAGAATATTGTGCAAC-3' |
|  |  | glpQ-BM-R2 | 5'-CATTACTGTGTCAGTAAAATCTGTAAATATACCATCTAC-3' |
| *Babesia species* | *18S rRNA* | Bath-F | 5'-TAAGAATTTCACCTCTGACAGTTA-3' |
|  |  | Bath-R | 5'-ACACAGGGAGGTAGTGACAAG-3' |
